# A Simple Method for Fabricating an External Light Extraction Composite Layer with RNS to Improve the Optical Properties of OLEDs

**DOI:** 10.3390/nano12091430

**Published:** 2022-04-22

**Authors:** Geun-Su Choi, Shin-Woo Kang, Eun-Jeong Bae, Eun-Bi Jang, Dong-Hyun Baek, Byeong-Kwon Ju, Young-Wook Park

**Affiliations:** 1 Nano and Organic-Electronics Laboratory, Department of Display and Semiconductor Engineering, Sun Moon University, Asan 31460, Korea; crs4964@sunmoon.ac.kr (G.-S.C.); newoosw@korea.ac.kr (S.-W.K.); baeej2@sunmoon.ac.kr (E.-J.B.); kksk0428@sunmoon.ac.kr (E.-B.J.); 2 Display and Nanosystem Laboratory, Department of Electrical Engineering, Korea University, Seoul 02841, Korea; 3 Center for Next Generation Semiconductor Technology, Department of Display and Semiconductor Engineering, Sun Moon University, Asan 31460, Korea; dhbaek@sunmoon.ac.kr

**Keywords:** organic light-emitting diodes, reactive ion etching, random nanostructure, nanoparticle, scattering effect, external light extraction

## Abstract

In this study, we fabricated a random nanostructure (RNS) external light extraction composite layer containing high-refractive-index nanoparticles through a simple and inexpensive solution process and a low-temperature mask-free process. We focused on varying the shape and density of the RNSs and adjusted the concentration of the high-refractive-index nanoparticles to control the optical properties. The RNSs fabricated using a low-temperature mask-free process can use the distance between the nanostructures and various forms to control the diffraction and scattering effects in the visible light wavelength range. Consequently, our film exhibited a direct transmittance of ~85% at a wavelength of 550 nm. Furthermore, when the RNSs’ composite film, manufactured using the low-temperature mask-free process, was applied to organic light-emitting diodes (OLEDs), it exhibited an external quantum efficiency improvement of 32.2% compared with the OLEDs without the RNSs. Therefore, the randomly distributed high-refractive-index nanoparticles on the polymer film can reduce the waveguide mode and total reflection at the substrate/air interface. These films can be used as a scattering layer to reduce the loss of the OLED substrate mode.

## 1. Introduction

Organic light-emitting diodes (OLEDs) are widely applied to mobile phone and television displays due to their self-emission characteristics, excellent color gamut, and high operating speed. However, the external quantum efficiency of OLEDs is approximately 20%. The surface plasmon polariton mode loses light at the metal–organic interface, the waveguide mode at the indium tin oxide (ITO)/organic layer, and the substrate mode at the glass substrate [1,2,3]. In general, the refractive index difference between the ITO/glass interface and the glass/air interface is slight, so the light loss in the substrate mode is greater than the light loss in the waveguide mode [4]. Therefore, researchers have attempted to find solutions to improve OLEDs’ light extraction and viewing angle [5]. Internal, external, and internal–external light extraction films have been adopted to improve light extraction and viewing angles, including microlens arrays (MLA), nanopores, convex structures, concave structures, low refractive index gratings, and structures composed of pores [6,7,8,9]. Among them, the external light extraction film is a technology that must be applied to the outside of the device and can be manufactured regardless of the shape of the device. The method of modulating the internal or external film of the OLED substrate minimizes the total internal reflection of the device. These films are formed by various techniques, including photolithography, printing, and molding. However, these techniques use at least lithography templates. Therefore, it is complicated or expensive for mass production. However, other researchers have reported superficial scattering layers synthesized in a competitive, scalable way. A hemispherical MLA was used as the scattering layer in this method. Yue Qu et al. reported that they fabricated a green OLED device that represents a 65% enhancement of external quantum efficiency by a high-refractive index, a hemispherical MLA built into a glass substrate [10]. However, because the lenses are microscale or the pitch between the lenses is microscale, these techniques exhibit small diffraction effects that are difficult to control. Moreover, to prevent the loss of the substrate mode, the most-represented MLA has larger visibility than a nano-sized array because of its size.

We propose a simple method to manufacture an RNS as a scattering layer to enhance the light extraction of OLEDs and improve the viewing-angle characteristics. In the fluorescent OLED devices fabricated in this study, the Lambertian distribution increased according to the change in the viewing angle. This scattering layer is fabricated through etching at low temperatures without spin coating and photomasking. It is fabricated as a cost-effective and scalable procedure. RNSs fabricated using a low-temperature mask-free process can use the distance between the nanostructures and various forms to control diffraction and scattering effects in the visible light wavelength range. Consequently, our film exhibited a perpendicular transmittance of ~85% at a wavelength of 550 nm. Also, when the RNS composite film manufactured using the low-temperature mask-free process was applied to OLEDs, it exhibited an external quantum efficiency improvement of 32.2% compared with the OLEDs without the RNSs.

## 2. Materials and Methods

**Figure 1:**
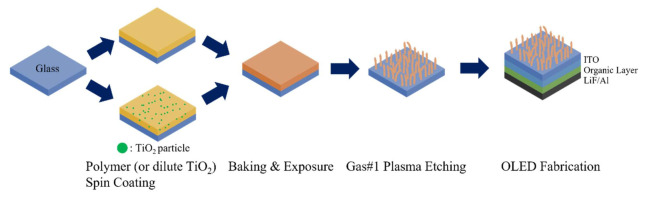
Schematic of the OLED fabrication process with RNSs (or diluted TiO_2_).

Figure 1 shows the fabrication process of the scattering layer. First, SU-8 (or dilute TiO_2_) was spin-coated onto the cleaned glass substrate at a rotational speed of 1500 rpm, baked at a 95 °C hot plate for 2 min, and exposed at 240 mJ/cm^2^ to conventional UV radiation (350–400 nm). The film was etched in a reactive ion etcher (RIE) using oxygen plasma at the RF power of 200 W, process pressure of 32 mTorr, and gas flow of 20 sccm [11]. After the first etching of the polymer, the structures were random and columnar-like, with high aspect ratios and densities. To investigate the properties of the RNSs, organic light-emitting devices were fabricated. External light extraction films altered the duration of oxygen plasma treatment to obtain devices with different properties, whereas the other conditions were unchanged. After the RNSs were formed on the glass substrate, the following layers were then thermally evaporated onto the ITO under vacuum conditions (~10^−7^ Torr): a 100 nm thick N, N′-Bis(naphthalen-1-yl)-N, N′-bis(phenyl) benzidine (NPB) as the hole-transport layer, a 40 nm thick Tris-(8-hydroxyquinoline)aluminum (Alq_3_) as the emission and electron-transport layers, a 1.0 nm thick lithium fluoride layer for electron injection, and a 150 nm thick aluminum layer that acted as the cathode.

## 3. Results and Discussion

**Figure 2:**
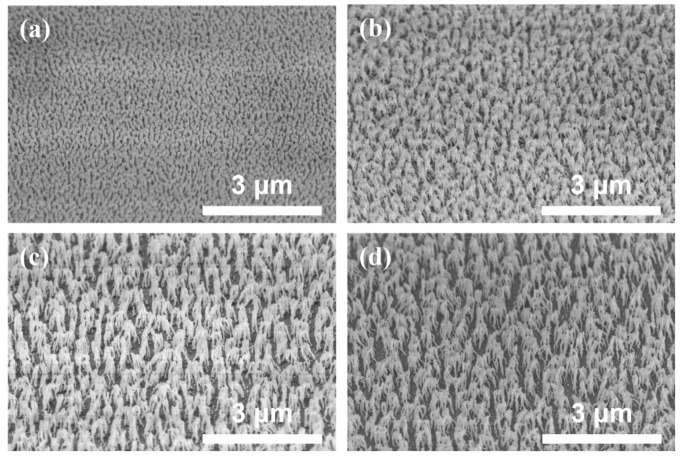
Cross-sectional scanning electron microscopy images (tilt angle of 45°) of random nanostructures (RNSs) obtained under different plasma-etching conditions: (**a**) RNSs 1-1, (**b**) RNSs 1-2, (**c**) RNSs 1-3, and (**d**) RNSs 1-4.

It is desirable to fabricate a suitable height and periodicity nanostructure comparable to the visible range wavelength to enhance light extraction. For manufacturing RNSs, SU-8 polymer (or dilute TiO_2_) was chosen because it is highly transparent to visible light and has a higher refractive index than glass. The RNSs were obtained using only oxygen (O_2_) plasma for a duration of 120, 240, 480, and 720 s, respectively (denoted as RNSs 1-1, RNSs 1-2, RNSs 1-3, and RNSs 1-4, respectively). The oxygen plasma process pressure remained unchanged. The structure was random and columnar-like with a high aspect ratio and density (Figure 2a–d) [12]. After the first etching, the structure randomly formed rod shapes with a high aspect ratio and density. To maximize light outcoupling, selecting a nanosized structure size and periodicity comparable to the wavelength of light is desirable. We changed an additional O_2_ plasma treatment duration to control the height and density.

**Figure 3:**
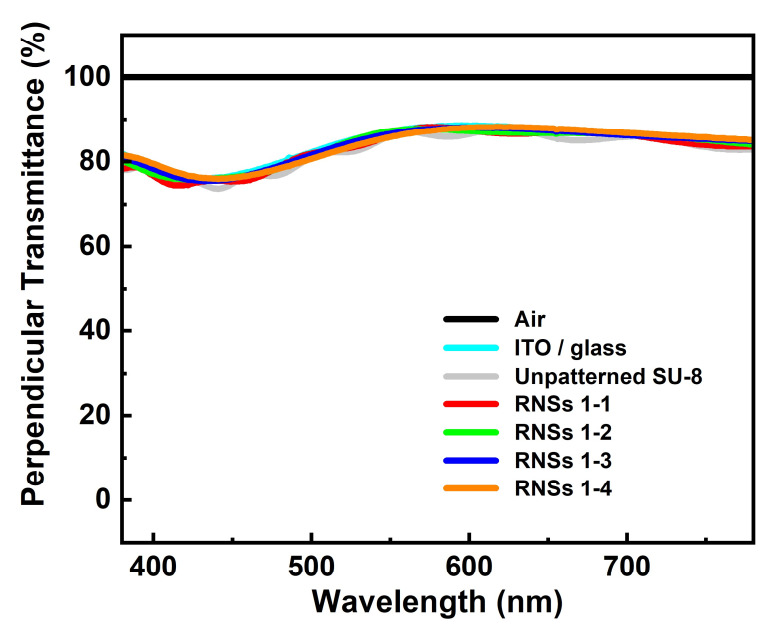
Perpendicular visible wavelength transmittance of different patterned polymers. RNSs 1-1, RNSs 1-2, RNSs 1-3, and RNSs 1-4.

To confirm the scattering effect, we measured the perpendicular transmittance. As shown in Figure 3, the perpendicular transmittance of RNSs, comprising only the SU-8 structure, is higher than 85% in the visible region because SU-8 is a transparent polymer material with a transmittance of 90% or more in the visible region. The perpendicular transmittance values in the green wavelength region (at 550 nm) used in this paper were 86.1, 87.2, 86.5, and 85.8% in RNSs 1-1, RNSs 1-2, RNSs 1-3, and RNSs 1-4, respectively.

**Figure 4:**
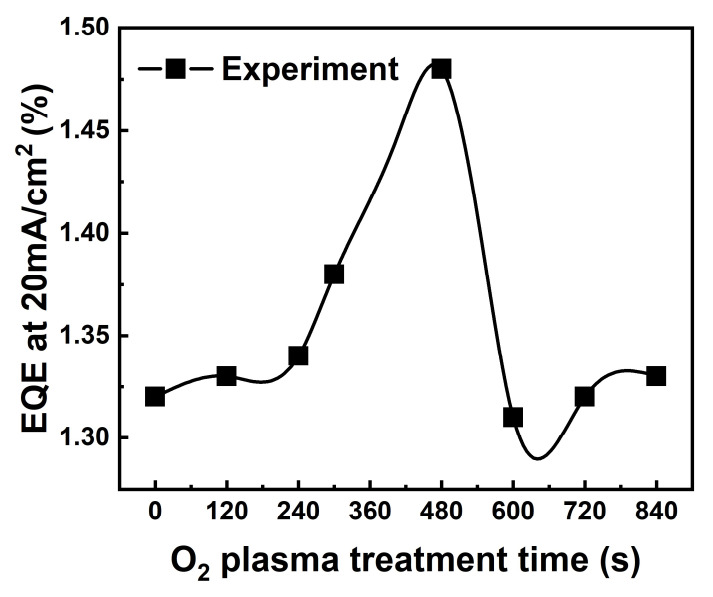
EQE characteristics as a function of current density: The OLEDs fabricated with varying the O_2_ plasma treatment duration to analyze the effect of the external light extraction film on the device characteristics.

We confirmed the effect of applying the scattering layers fabricated in this way to actual OLED devices. OLEDs were fabricated to investigate the properties of RNSs. First, the scattering layers were applied to the outside of the OLEDs of the conventional structure using the transparent ITO electrode as the anode and the characteristics were evaluated. Figure 4 shows the summarized external quantum efficiency (EQE) characteristics of the OLEDs fabricated with varying the O_2_ plasma treatment duration to analyze the effect of the external light extraction film on the device characteristics. The in-detail EL characteristics, including the current density-voltage and others, are presented in the Supplementary Materials (Figure S1). The height and density modulation of the nanostructures in the external light extraction film is a necessary process to improve the light extraction efficiency of the OLEDs [13,14,15,16]. To modulate the height and density of nanorods, OLEDs were fabricated by modulating the O_2_ plasma treatment duration from 0 to 840 s. As the O_2_ plasma treatment duration increased, the height of the RNSs increased and it was confirmed that EQE was improved up to 480 s. However, when the O_2_ plasma treatment duration was longer than a certain level, it was confirmed that the EQE was lowered as the height of the RNSs was reduced. The device showed the highest EQE at 480 s (EQE at 20 mJ/cm^2^ was 1.48%). As previously reported, we confirmed that the nanostructures improved EQE [17,18,19]. From these results, we conclude that the nanostructures fabricated with an O_2_ plasma treatment duration of 480 s lead to high light-extraction efficiency due to the scattering and diffraction effects.

**Figure 5:**
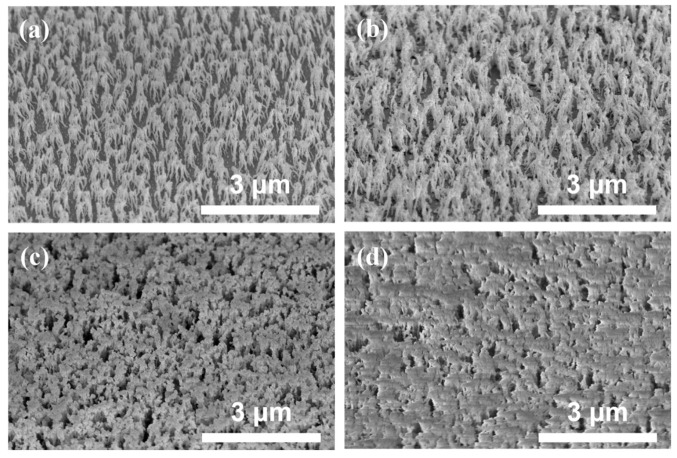
Cross-sectional (tilt angle of 45°) SEM images of diffusion layer: (**a**) RNSs 1-3, (**b**–**d**) TiO_2_-diluted RNSs 1-3-1, 1-3-2, and 1-3-3 (1.0, 4.0, and 8.0 wt%, respectively).

Then, a composite film in which TiO_2_ was diluted to various weight percentages (%) in the RNSs was prepared to improve the light-extraction structure further. Figure 5 shows the scanning electron microscope (SEM) and cross-sectional image of the light-extraction film surfaces. The TiO_2_ nanoparticles are embedded into SU-8 under various conditions (weight percent: 1.0–8.0 wt%) of a mixed SU-8 + TiO_2_ solution. As confirmed by the SEM image, the number of cone-shaped pillars increases as the weight percent of TiO_2_ increases in the SU-8 + TiO_2_ mixed solution.

**Figure 6:**
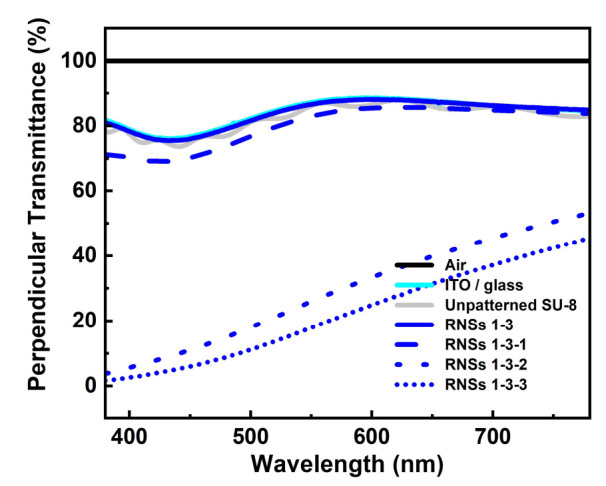
Visible light wavelength perpendicular transmittance of different TiO_2_ diluted RNSs.

To confirm the scattering effect of the composite film, we measured the perpendicular transmittance. As shown in Figure 6, the perpendicular transmittance values in the green wavelength region (at 550 nm) were 86.5, 83.0, 25.9, and 17.9%, respectively. (RNSs 1-3, RNSs 1-3-1, RNSs 1-3-2, and RNSs 1-3-3, respectively) Conversely, the TiO_2_-diluted RNSs show lower perpendicular transmittance values than the RNSs. This decrease in transmittance is presumably due to the transmittance of TiO_2_ at the top.

**Figure 7:**
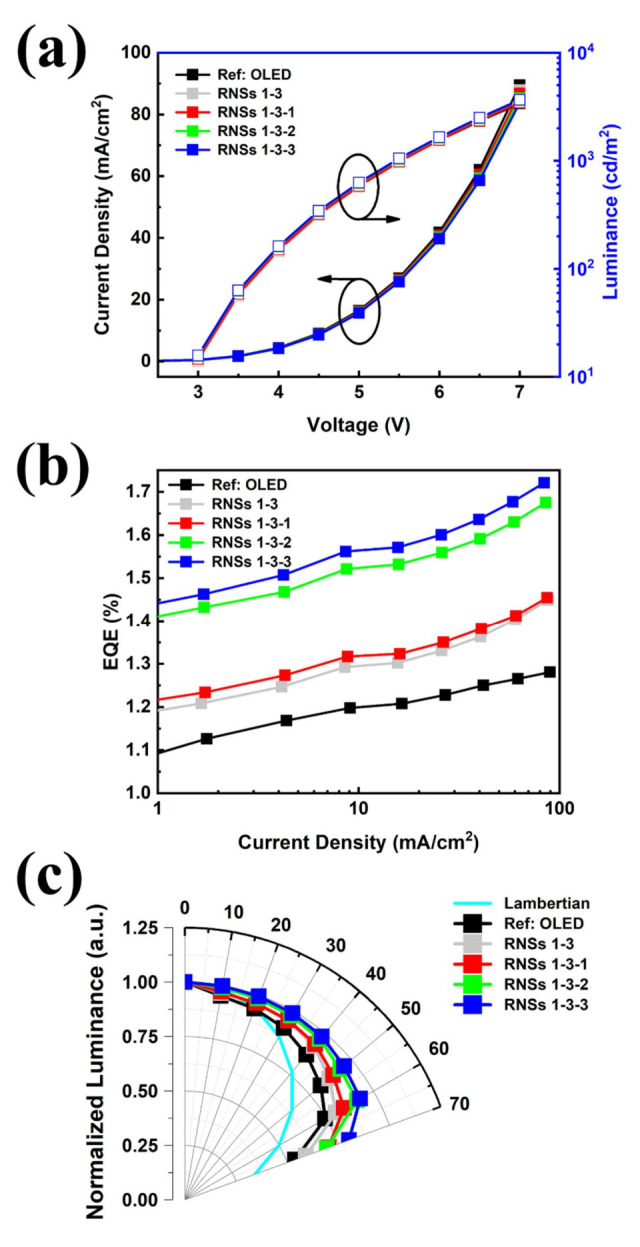
Comparison of light efficiency characteristics of scattering layers in which TiO_2_-diluted RNSs are applied to OLED devices. (**a**) Current density-voltage characteristics of devices, (**b**) external quantum efficiency as a function of current density characteristics of fabricated OLEDs, and (**c**) normalized angular luminance distributions of OLEDs between 0° and 70°.

The device’s electrical characteristics both with and without the RNSs + TiO_2_ were almost identical from when they were placed and do not affect the devices (Figure 7a). The OLEDs with the RNSs + TiO_2_ exhibited higher efficiencies than those without the RNSs in terms of overall current density (Figure 7b). The OLEDs applied with the composite layer diluted to 8 wt% TiO_2_ showed the EQE at 20 mA/cm^2^ of 1.60%, whereas the reference device exhibited an EQE at 20 mA/cm^2^ of 1.21%. The enhancement in the EQE at 20 mA/cm^2^ was 32.2% in the normal direction. It is expected to induce low total reflection due to the high refractive index TiO_2_ nanoparticles (*n* > 2.3) embedded in the RNSs, which is expected to contribute to the improvement of light extraction. Figure 7c shows that the OLEDs with RNSs + TiO_2_ composite layers exhibited improved luminance intensities with changes in viewing angle from 0° to 70° due to the scattering effect. The luminance was measured with a current density of 4 mA/cm^2^. For the best conditions (TiO_2_ diluted 8.0 wt%) based on a viewing angle of 50 degrees, the improvement in the luminance was 17.8% relative to the reference device. Under the conditions of TiO_2_ diluted at 0.0, 1.0, and 4.0, the wt% normalized luminance intensity increased by 6.9%, 9.7%, and 15.0%, respectively. Therefore, it was confirmed that the efficiency and viewing angle improved by reducing the loss of the substrate mode based on the scattering characteristics of the RNSs + TiO_2_.

**Figure 8:**
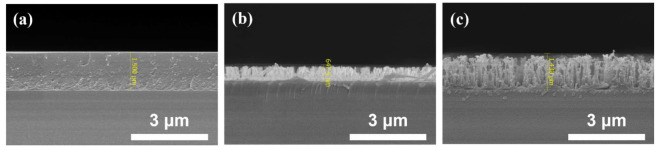
Cross-sectional scanning electron microscopy images of diffusion layer: (**a**) Unpatterned SU-8, (**b**) RNSs 1-3, and (**c**) RNSs 1-3-2.

This work demonstrated a simple method for fabricating RNSs with a scattering layer at low temperatures without a mask. As shown in Table 1, when O_2_ plasma was treated, it was confirmed that the overall height of the RNSs was lowered due to the etching characteristics (Figure 8b). On the other hand, when TiO_2_ is dispersed in the SU-8 film, it is distributed throughout the external light-extraction surface, thus preventing the etching of oxygen plasma and forming high-height RNSs (Figure 8c). In addition, it is assumed that TiO_2_, a high refractive material, will improve the scattering effect [20,21,22]. When TiO_2_ is dispersed in the SU-8 film, the perpendicular transmittance is lowered, but the total transmittance and haze are expected to increase as the EQE and viewing angle are widened. Through O_2_ plasma treatment on the SU-8 film, the EQE was improved when the RNSs were made. In addition, a greater improvement was confirmed when TiO_2_ was dispersed in SU-8.

## 4. Conclusions

We have shown a simple way to produce RNSs as a scattering layer at low temperatures without the need for a mask. In particular, the height and density of the RNSs can be controlled using the etching characteristics of oxygen plasma. Consequently, when the RNSs’ composite film fabricated using the low-temperature mask-free process was applied to OLEDs, it exhibited an external quantum efficiency improved by 32.2% compared with the OLEDs without the RNSs. By using these RNSs, it is possible to reduce variations in light intensity due to the viewing angle. In addition, high-refractive-index TiO_2_ nanoparticles are expected to have a lower total internal reflection due to the reduced angle of incidence, which may contribute to improved light extraction. This paper successfully demonstrated TiO_2_ composite nano light extraction technology. However, if SU-8 is coated more heavily and the duration of O_2_ plasma treatment is prolonged to fabricate tall RNSs, it is expected to achieve a higher level of improvement. This result has not been fully optimized, and higher efficiency is expected.

## Figures and Tables

**Table 1:** Average height of film, perpendicular transmittance, and the enhancement in EQE for different RNSs.

	Unpatterned SU-8	RNSs 1-3	RNSs 1-3-2
Average height of film (nm)	1500	647.5	1430
Perpendicular transmittance at 550 nm (%)	86.1	86.5	25.9
Enhancement in EQE (%)	0	12.9	28.9

## Data Availability

Not applicable.

## Supplementary Materials

The following supporting information can be downloaded at: https://www.mdpi.com/article/10.3390/nano12091430/s1, Figure S1: Comparison of luminous efficiency characteristics of scattering layers in which RNSs are applied to OLED devices. (a) current density-voltage characteristics of devices, (b) external quantum efficiency as a function of current density characteristics of fabricated OLEDs, (c) normalized angular luminance distributions of OLEDs between 0 ° and 70 °.
