# Supplementary material for: A Simple Method for Fabricating an External Light Extraction Composite Layer with RNS to Improve the Optical Properties of OLEDs
Source: Nanomaterials (Basel). 2022 Apr 22;12(9):1430. doi: 10.3390/nano12091430 (PMC9103064; doi:10.3390/nano12091430)
Supplement: Supplementary file 1 [file nanomaterials-12-01430-s001.zip › nanomaterials-1636434-supplementary.pdf]

Supporting Information

A Simple Method for Fabricating an External Light Extraction Composite Layer with RNS to Improve the Optical Properties of OLEDs

Geun-Su Choi [1], Shin-Woo Kang [1,2], Eun-Jeong Bae [1,2], Eun-Bi Jang [1], Dong-Hyun Baek [3] , Byeong-Kwon Ju [2,*] and Young-Wook Park [1,*]

[1] Nano and Organic-Electronics Laboratory, Department of Display and Semiconductor Engineering,
Sun Moon University, Asan 31460, Korea; crs4964@sunmoon.ac.kr (G.-S.C.); newoosw@korea.ac.kr
(S.-W.K.); baeej2@sunmoon.ac.kr (E.-J.B.); kksk0428@sunmoon.ac.kr (E.-B.J.)

[2] Display and Nanosystem Laboratory, Department of Electrical Engineering, Korea University,
Seoul 02841, Korea

[3] Center for Next Generation Semiconductor Technology, Department of Display and Semiconductor
Engineering, Sun Moon University, Asan 31460, Korea; dhbaek@sunmoon.ac.kr

* Correspondence: bkju@korea.ac.kr (B.-K.J.); zerook@sunmoon.ac.kr (Y.-W.P.)

1. EL characteristics of fluorescence OLEDs for various the O_2 plasma treatment duration

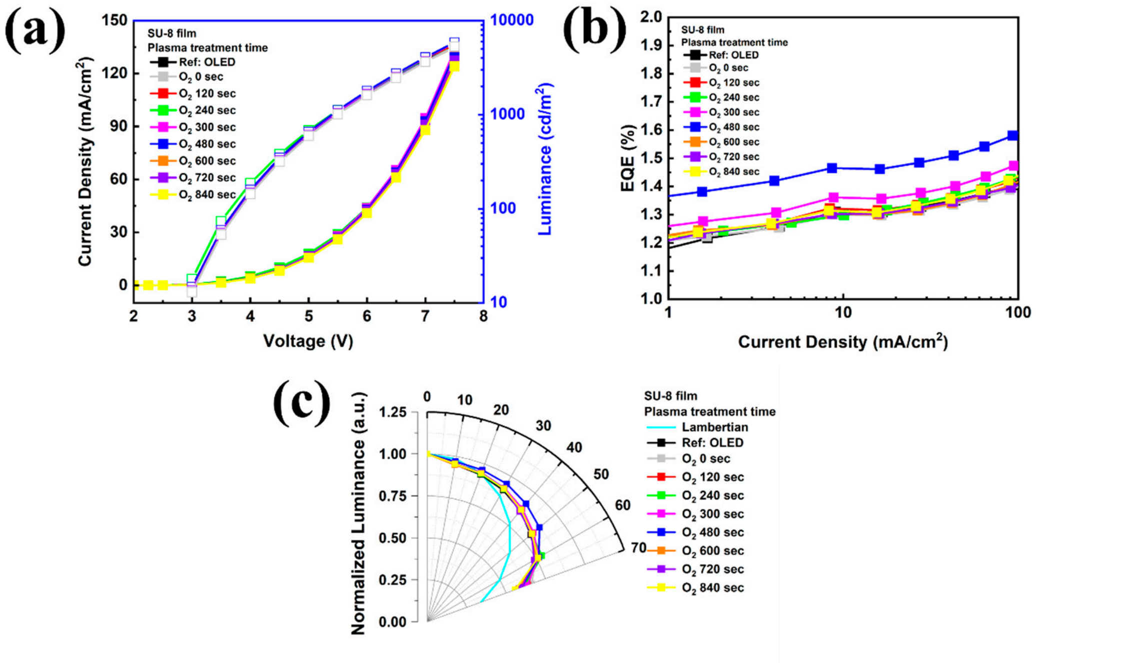

Figure S1. Comparison of luminous efficiency characteristics of scattering layers in which RNSs are applied to OLED devices. (**a**) current density-voltage characteristics of devices, (**b**) external quantum efficiency as a function of current density characteristics of fabricated OLEDs, (**c**) normalized angular luminance distributions of OLEDs between 0° and 70°.

The device's electrical characteristics with and without the RNSs were almost identical since they were placed, where they do not affect the devices (figure S1 (a)). The OLEDs with the RNSs

exhibited higher efficiencies than those without the RNSs in terms of overall current density. The OLED treated with oxygen plasma for 480 sec showed the maximum EQE at 20 mA/cm^2 of 1.48 %, while the reference device exhibited an EQE at 20 mA/cm^2 of 1.31 %. The enhancement in the EQE at 20 mA/cm^2 was 13.0 % in the normal direction (figure S1 (b)). Figure S1 (c) shows that the OLEDs with RNSs exhibited improved luminance intensities with changes in viewing angle from 0 ° to 70 ° due to the scattering effect. For the best conditions (O_2 plasma 480 s) based on a viewing angle of 50 degrees, the improvement in the luminance was 8.4 % relative to the reference device. Therefore, it was confirmed that the efficiency and viewing angle improved by reducing the loss of the substrate mode based on the scattering characteristics of the RNSs.
